# Evaluation of Cervical Cancer Screening Programs in Côte d’Ivoire, Guyana, and Tanzania: Effect of HIV Status

**DOI:** 10.1371/journal.pone.0139242

**Published:** 2015-09-25

**Authors:** Jean Anderson, Megan Wysong, Deb Estep, Giulia Besana, Sharon Kibwana, John Varallo, Kai Sun, Enriquito Lu

**Affiliations:** 1 Johns Hopkins Medical Institutions, Baltimore, Maryland, United States of America; 2 Jhpiego, an affiliate of Johns Hopkins University, Baltimore, Maryland, United States of America; 3 Jhpiego/Tanzania, an affiliate of Johns Hopkins University, Dar es Salaam, Tanzania; National Cancer Institute, UNITED STATES

## Abstract

**Background:**

HIV infection increases a woman’s risk for cervical cancer, and cervical cancer incidence and mortality rates are higher in countries with high HIV prevalence and limited resources for screening. Visual inspection with acetic acid (VIA) allows screening and treatment of cervical lesions in a single-visit approach (SVA), but data on its performance in HIV-infected women are limited. This study’s objective was to examine cervical cancer screening using VIA/SVA in programs serving HIV-infected women.

**Methods:**

A VIA/SVA program with cryotherapy for VIA-positive lesions was implemented in Côte d’Ivoire, Guyana, and Tanzania from 2009 to 2012. The effect of HIV status on VIA positivity and on presence of cryotherapy-eligible lesions was examined using a cross-sectional study design, with Chi-square tests for comparisons and constructed multivariate logistic regression models. A P-value of < 0.05 was significant.

**Findings:**

VIA was performed on 34,921 women, 10% (3,580) were VIA positive; 2,508 (85%) eligible women received cryotherapy during the same visit; only 234 (52%) of those who postponed returned for treatment; 622 (17%) VIA-positive women had lesions too large to be treated with cryotherapy and were referred for excisional treatment. In multivariate analysis—controlling for HIV status, location of the screening clinic, facility location, facility type, and country—compared to HIV-uninfected/unknown women, HIV-infected women had higher odds of being VIA positive (OR 1.95, 95% CI 1.76, 2.16, P<0.0001) and of having large lesions requiring referral (OR 1.93, 95% CI 1.49, 2.51, P< 0.0001). Minor treatment complications occurred in 19 of 3,032 (0.63%) women; none required further intervention.

**Conclusions:**

This study found that compared to HIV-uninfected/unknown women, HIV-infected women had nearly twice the odds of being VIA-positive and to require referral for large lesions. SVA was safe and resulted in significant reductions in loss to follow-up. There is increased need for excisional treatment in countries with high HIV prevalence.

## Introduction

Compared to HIV-negative women, women infected with HIV have higher prevalence rates and longer persistence of human papillomavirus (HPV) infection, the primary cause of cervical cancer, and higher rates of cervical dysplasia, the precursor of invasive cervical cancer.[1,2] Furthermore, cervical cancer incidence and mortality rates are higher in countries with high HIV prevalence rates and few resources for screening and prevention.[3,4]

Because invasive cervical cancer does not develop until approximately 10–15 years after initial HPV infection, there is an opportunity to diagnose and treat cervical cancer precursors and to interrupt progression to cancer.[5] Since the introduction of cervical cytology, mortality from cervical cancer in developed countries has decreased by more than 70%. The lifetime risk of cervical cancer can be reduced by approximately 80–90% by screening women every three to five years.[6] However, in countries with limited resources, implementation of cervical cytology services is constrained by the inadequate health infrastructure, including a lack of cytopathologists and cytology technicians to prepare and analyze Pap smears, and the need for follow-up visits for further evaluation and treatment when Pap smears are abnormal.[7]

Visual inspection of the cervix with acetic acid (VIA) is a low-cost, low-technology approach to cervical cancer screening that enables identification of precancerous lesions that can be treated with ablative (e.g., cryotherapy) or excisional (e.g., loop electrosurgical excision procedure, or LEEP) treatment, potentially in a single-visit approach (SVA). VIA/SVA has been shown to be a cost-effective, safe, feasible, and acceptable alternative to cytology, with comparable sensitivity.[8,9]

However, there are limited data on the provision, performance, and integration of cervical cancer screening using VIA/SVA in programs serving women with HIV infection and how findings may differ from HIV-uninfected women. This paper reports results from three country programs that introduced VIA/SVA for cervical cancer screening and prevention into already existing private and public sector clinics, with analysis of findings by HIV status.

## Materials and Methods

### Design

This study reports results from a program evaluation involving three countries (Guyana in South America, Côte d’Ivoire in West Africa, and Tanzania in East Africa) that implemented a cervical cancer screening program using VIA/SVA from January 2009 to March 2012 in 24 HIV care and treatment and 23 reproductive and child health clinics (Table 1). Using information from first screening appointments, the effect of HIV status on VIA positivity and on presence of large, cryotherapy-ineligible lesions requiring referral, was examined using a cross-sectional study design.

**Table 1:** Baseline facility and provider characteristics, by country.

Variables	Côte d’Ivoire	Guyana	Tanzania	Total
Service Delivery Period				
Start date–end date	Oct 2009 –Mar 2012	Jan 2009 –Mar 2012	Apr 2010 –Mar 2012	Jan 2009 –Mar 2012
**Number of Project Sites**				
Total	20	16	11	47
**Location of Cervical Cancer Screening Clinic**				
HIV care and treatment clinic (%)	20 (100%)	4 (25%)	0 (0%)	24 (51%)
Reproductive and child health clinic (%)	0 (0%)	12 (75%)	11 (100%)	23 (49%)
**Facility Location**				
Urban (%)	20 (100%)	6 (38%)	2 (18%)	28 (60%)
Peri-urban (%)	0 (0%)	2 (13%)	2 (9%)	4 (9%)
Rural (%)	0 (0%)	8 (50%)	7 (64%)	15 (32%)
**Facility Type**				
National hospitals (%)	1 (5%)	1 (6%)	0 (0%)	2 (4%)
Regional hospital (%)	4 (20%)	7 (44%)	2 (18%)	13 (28%)
District hospital (%)	9 (45%)	5 (31%)	7 (64%)	21 (45%)
Health center (%)	6 (30%)	3 (19%)	2 (18%)	11 (23%)
**Number of people trained**				
Total	108	43	34	185
**Cadre of trained cervical cancer screening providers**				
Physician/clinical officers, medical officers/medex (%)	52 (48%)	27 (63%)	11 (32%)	90 (49%)
Nurse (%)	12 (11%)	9 (21%)	23 (68%)	44 (24%)
Midwife (%)	44 (41%)	7 (16%)	0 (0%)	51 (27%)
Age of provider (years)				
Average (range)	41 (27–55)	40 (25–55)	42 (26–61)	41 (25–61)

### Procedures and Participants

To prepare providers to perform VIA and cryotherapy, all three countries used a standardized package that consisted of six days of training covering basic information about cervical cancer and its precursors and included a practicum component and a skills/knowledge assessment. Providers’ competency was assessed by an objective test of knowledge and by trainers’ observations of their performance of procedures. The providers were nurses, midwives, midlevel providers (clinical officers, assistant medical officers, and medex—a nationally recognized cadre of midlevel-providers in Guyana), and physicians.

Prior to VIA, all women received education and counseling. VIA was performed by application of 3–5% acetic acid to the cervix; one minute after application the cervix was examined with the naked eye using a bright light. With visual inspection techniques, there are three possible results: negative, positive, or suspicious for cancer, with the last result requiring referral and further evaluation and management. VIA was considered negative when no acetowhite lesions were detected; positive when dense, opaque, well-defined acetowhite lesions involving the squamocolumnar junction were seen; and suspicious for cancer if a friable exophytic mass or ulcerative lesion was present on initial visualization. Women who were VIA negative were given a card with their diagnosis and a recommendation to be rescreened in three to five years if HIV-uninfected and in one year if HIV-infected. Women with a positive VIA result were offered immediate cryotherapy when all of the following criteria were met: the lesion involved less than three quadrants of the transformation zone with no extension greater than two millimeters into the cervical canal or extension onto the vaginal walls; the entire lesion could be covered by the cryoprobe; the squamocolumnar junction was fully visible; and there was no suspicion of invasive cancer.

Cryotherapy was done with a double-freeze technique (three-minute freeze, five-minute thaw, three-minute freeze) without local anesthesia. After screening and/or treatment, women were given a card with their diagnosis and a recommended follow-up date. Women were advised on precautions after cryotherapy, including avoiding sexual activity for four weeks during healing, and symptoms of possible complications (e.g., bleeding, infection). Condom use was recommended for all sexual activity. Women were advised to return for clinical follow-up one year after treatment, for repeat VIA to assess treatment success. Women with lesions that were not eligible for cryotherapy were referred to a site that offered LEEP; those with lesions suspicious for invasive cancer were referred for confirmatory diagnosis and definitive treatment. Provider competency was maintained by supervision in the field on at least a quarterly basis, with direct observation and extended onsite coaching and mentoring.

In this study, VIA/SVA programs targeted but were not limited to HIV-infected women between the ages of 30 and 50 who self-selected to receive screening. Oral informed consent for screening was given by all women, and women receiving treatment gave consent again at the time of the treatment. HIV status was by self-report in Guyana and by record review for Tanzania and Côte d’Ivoire. Patients were considered to have HIV-unknown status if they reported never receiving HIV testing or results of testing. Provider-initiated HIV testing and counseling was also integrated into the cervical cancer screening program in Tanzania.[10] Positive HIV status was confirmed by review of patient cards carried to clinical visits when available. Patient-level information regarding CD4 count and antiretroviral therapy (ART) was not available for this analysis. Providers were not blinded to the HIV status of the patient prior to screening.

### Statistical Analysis

For Côte d’Ivoire and Guyana, aggregate data by facility were collected using a monthly summary form with 10 key binary indicators reflecting outcomes of screening, treatment, and referral, disaggregated by HIV status and visit type (initial visit, routine screening after negative result at first screening, and follow-up visit one year after treatment), and entered into Excel. There were no individual patient identifiers. In Tanzania, patient-level data from individual client records were entered into a database. All data were de-identified and all patient records and information were anonymized and de-identified by data entry personnel prior to sending it to the authors for analysis. The aggregated data were converted into individual data first and then the data for the three countries were analyzed separately and then pooled for the analysis. For this analysis, women who reported HIV negative status and those of unknown status were grouped together, as they could not be reliably separated due to the aggregation configuration of the monthly reporting format used in Guyana and Cote d’Ivoire, problems with consistent availability of HIV testing in Tanzania, and, for those reporting HIV negative results, the variable time interval since testing. Variables were reported as percentages and chi-square tests were performed. Multivariable logistic regression models were used to examine the relationship between HIV status and VIA status and between HIV status and larger lesions status of VIA-positive patients. Variables that were associated with VIA status at a level of significance of P < 0.2 were included in the multivariable analyses as potential confounders. The statistical program used was SAS Version 9.3 (SAS Institute, Cary, NC). The overall level of significance used in this study was P < 0.05.

This program evaluation protocol was reviewed and determined to not qualify as human subjects research as defined by DHHS regulations 45 CFR 46.102 by the Johns Hopkins Bloomberg School of Public Health Institutional Review Board and each country’s Ministry of Health determined that this was public health practice data and not research so no local institutional review board application was necessary after the MOH approval.

## Results

### Baseline Characteristics

Baseline characteristics of facilities and providers are presented by country in Table 1. All facilities in Côte d’Ivoire were HIV care and treatment sites, although some women of unknown or uninfected HIV status were also seen in these sites. In all sites in Tanzania and in 75% of Guyana sites, services were provided in reproductive and child health clinics, and HIV-infected women in care were referred from a co-located HIV care and treatment clinic. Urban, peri-urban, and rural sites were well-represented in Tanzania and Guyana; in Côte d’Ivoire, all 20 sites were classified as urban. All but one facility in Guyana were part of the public health system. A total of 185 providers were trained in and provided VIA/SVA services; 51% were nurses or midwives.

### Overall Project Results

**Fig 1:**
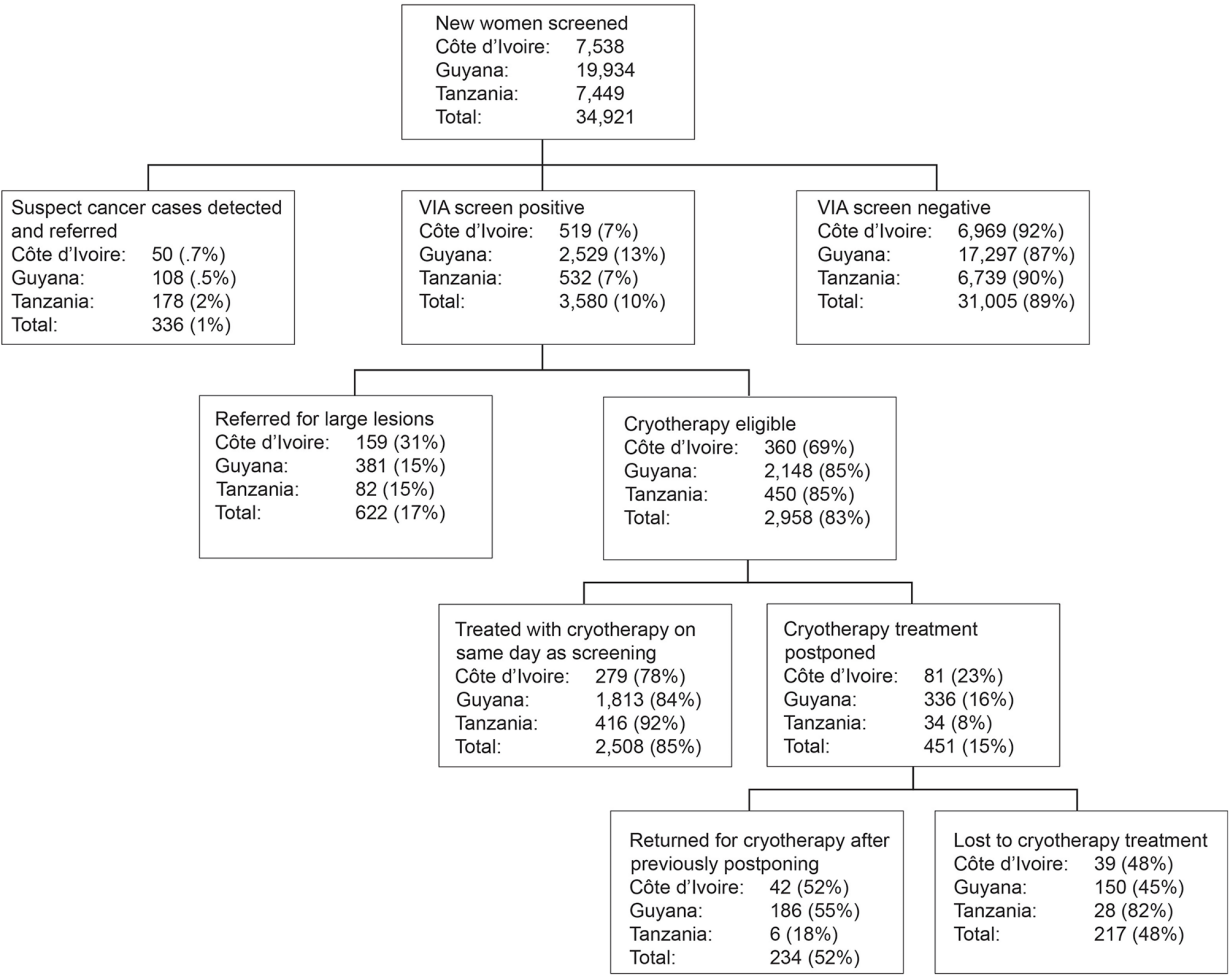
Cervical cancer screening and treatment outcomes in three countries.

Fig 1 presents cumulative results of key indicators by country from the beginning of the project through the end of data collection for this report. A total of 34,921 women received VIA screening for the first time between January 2009 and March 2012, and VIA-positive rates ranged from 7% to 13% in the three countries (mean 10%). Cases suspicious for invasive cancer were 2% or less for all cases in Côte d’Ivoire, Guyana, and Tanzania. Across the three countries an average of 85% (range of 78–92%) of VIA-positive women who were eligible for cryotherapy received this procedure at the same visit. Among those who postponed treatment, only 52% (range of 18–55%) returned later for cryotherapy. A total of 622 (17%) of the VIA-positive women (range of 15–31%) had lesions that were too large to be treated effectively with cryotherapy. These women were referred for LEEP, although capacity to perform LEEP was limited in all three countries during the time covered by this analysis and only 290 (47%) LEEPs were performed. LEEP was not performed in a single-visit approach in any of the three countries. Both issues contributed to a high loss-to-follow-up rate in each country (data not shown).

### Effect of HIV Status

In each country, HIV-infected women were more likely to be VIA-positive and were more likely to have large lesions that were not eligible for treatment with cryotherapy (Table 2) than uninfected/unknown women. In bivariate analysis, variables significantly associated with VIA positivity included location of the screening, facility type, and country, but not HIV status or facility location (Table 3). However, with multivariate analysis, adjusted for each of these variables, found HIV infection to be an independent predictor of VIA positivity. This effect was modified significantly by the country when compared with women who were HIV-uninfected or with unknown HIV status (Guyana OR 1.32, 95% CI 1.14, 1.54, P 0.0003; Côte d’Ivoire OR 3.33, 95% CI 2.44, 4.52, P <0.0001; Tanzania OR 2.52, 95% CI 2.09, 3.03, P <0.0001) Overall, HIV-infected women in all three countries had 1.95 times higher odds of being VIA-positive than women without HIV or with unknown HIV status (95% CI 1.76, 2.16, P < 0.0001) (Table 3). In the logistic regression model, women undergoing VIA in Guyana had higher odds of being VIA-positive as compared to Tanzania (Guyana OR 2.22, 95% CI 1.97, 2.51, P<0.0001). Being seen in an HIV clinic was associated with lower likelihood of VIA-positivity (HIV clinic OR 0.75, CI 0.64, 0.89, P.001).

**Table 2:** Comparison of VIA and large lesion results in new women screened, by country and HIV status, January 2009 –March 2012.

Screening Result, by Country	Total N = 34,921	HIV Positive N = 9,181	HIV negative/ unknown N = 25,740	P-value
**Côte d’Ivoire**	n = 7,538	n = 5,782	n = 1,756	
VIA negative	6,969 (92%)	5,268 (91%)	1,701 (97%)	< 0.0001
VIA positive	519 (7%)	473 (8%)	46 (3%)	< 0.0001
Large lesions	159 (31%)	152 (32%)	7 (15%)	0.0175
Cryotherapy eligible	360 (69%)	321 (68%)	39 (84%)	0.0175
Suspect cancer	50 (.7%)	41 (0.7%)	9 (0.5%)	0.3741
**Guyana**	n = 19,934	n = 1,602	n = 18,332	
VIA negative	17,297 (87%)	1,344 (84%)	15,953 (87%)	< 0.0001
VIA positive	2,529 (13%)	256 (16%)	2,273 (12%)	< 0.0001
Large lesions	381 (15%)	62 (24%)	319 (14%)	< 0.0001
Cryotherapy eligible	2,148 (85%)	194 (76%)	1,954 (86%)	< 0.0001
Suspect cancer	108 (.5%)	2 (0.1%)	106 (0.6%)	0.0178
**Tanzania**	n = 7,449	n = 1,797	n = 5,652	
VIA negative	6,739 (90%)	1,531 (85%)	5,208 (92%)	< 0.0001
VIA positive	532 (7%)	237 (13%)	295 (5%)	< 0.0001
Large lesions	82 (15%)	51 (22%)	31 (11%)	0.0005
Cryotherapy eligible	450 (85%)	186 (78%)	264 (89%)	0.0005
Suspect cancer	178 (2%)	29 (1.6%)	149 (2.6%)	0.0134

**Table 3:** Bivariate and multivariate logistic regression model of the odds of screening VIA positive, by HIV status and facility characteristics, all countries combined.*

Variables	VIA Positive (n = 3,580) (%)	VIA Negative (n = 31,005) (%)	P	OR	95% CI	P
**HIV Status**						
HIV Positive	27.0	26.3	.035	1.95	1.76, 2.16	<0.0001
HIV Negative/Unknown	73.0	73.7		(r)		
**Location of Cervical Cancer Screening Clinic**						
HIV care and treatment clinic	27.9	33.6	<0.0001	0.75	0.64, 0.89	.001
Reproductive and child health clinic	72.1	66.4		(r)		
**Facility Location**						
Urban	69.8	69.1	0.12	1	-	-
Per-urban	6.8	6.2		1.04	0.88, 1.24	0.63
Rural	23.4	24.7		0.99	0.86, 1.15	0.94
**Facility Type**						
National Hospital	35.0	30.7	<0.0001	.92	0.83, 1.02	0.12
Regional Hospital	20.6	18.6		(r)		
District Hospital	22.6	27.0		0.93	0.83, 1.03	0.17
Health Center	21.8	23.7		1.03	0.89, 1.20	0.69
**Country**						
Côte d’Ivoire	14.5	22.5	<0.0001	0.87	0.71, 1.07	0.18
Guyana	70.6	55.8		2.22	1.97, 2.51	<0.0001
Tanzania (r)	14.9	21.7		(r)		

(r) Reference category

* The total number of women included in this analysis is lower than the total number of women screened due to missing variables for some women, who were excluded from analysis.

When examining predictors of large lesions requiring referral among women who were VIA-positive, bivariate analysis found that HIV status, location of the screening, facility type and location, and country were all significantly associated with presence of large lesions (Table 4). However, multivariate logistic regression adjusting for each of these variables found that independent predictors of large lesion size included HIV status, location of VIA screening clinic and being screened in a national facility as compared to a regional facility (HIV status OR 1.93, CI 1.49, 2.51, P <0.0001; HIV clinic OR 3.44, CI 2.19, 5.42, P <0.0001; national facility OR 2.16, CI 1.57, 2.97, P, 0.0001). HIV status was not associated with increased odds of having lesions suspicious for cancer (data not shown).

**Table 4:** Bivariate and multivariate logistic regression model of the odds of having a large lesion diagnosed at the first VIA screening, by HIV status and facility characteristics, all countries combined.

Variables	Small Lesion (n = 2,958) (%)	Large Lesion (n = 622) (%)	P	OR	95% CI	P
**HIV Status**						
HIV Positive	42.6	23.7	<0.0001	1.93	1.49, 2.51	<0.0001
HIV Negative/Unknown	57.4	76.3		(r)		
**Location of Cervical Cancer Screening Clinic**						
HIV care and treatment clinic	43.8	24.6	<0.0001	3.44	2.19, 5.42	<0.0001
Reproductive and child health clinic	56.2	75.4		(r)		
**Facility Location**						
Urban	78.3	68.1	<0.0001	(r)		
Peri-urban	4.8	7.1		1.29	0.80, 2.07	0.57
Rural	16.9	24.8		1.30	0.90, 1.89	0.44
**Facility Type**						
National Hospital	35.9	34.8	<0.0001	2.16	1.57, 2.97	<0.0001
Regional Hospital	14.2	22.0		(r)		
District Hospital	21.6	22.8		0.93	0.65, 1.33	0.69
Health Center	28.3	20.4		0.84	0.57, 1.25	0.39
**Country**						
Côte d’Ivoire	25.6	12.2	<0.0001	0.56	0.35, 0.93	0.03
Guyana	61.2	72.6		0.72	0.51, 1.02	0.06
Tanzania	13.2	15.2		(r)		

(r) Reference category

Of all women who were VIA-positive and had lesions eligible for cryotherapy, 85% were treated at the same visit; of those who postponed treatment, only 52% returned for cryotherapy at a later date and there were no differences by HIV status (data not shown).

### Complications

Over the course of this study, 19 of 3,032 treated women returned (0.63%) to the health facility and reported treatment complications after treatment with either cryotherapy or LEEP (reporting in the case of a complication did not distinguish the type of procedure). All but one were HIV-uninfected women, and all cases were considered minor, not requiring referral or ongoing care (data not shown).

## Discussion

In this analysis from three countries with high HIV prevalence, HIV status was an independent predictor of VIA positivity, consistent with an increased prevalence of precancerous cervical lesions in the setting of HIV. Furthermore, women known to be HIV positive required referral for large lesions more frequently than women who were HIV uninfected/unknown. Overall, receiving immediate treatment in a single visit resulted in a significant reduction in loss to follow-up, compared to deferring treatment until a subsequent visit. There was no evidence of significant safety concerns with this approach in a low-resource setting or among HIV-infected women.

More than 85% of cervical cancer cases and deaths occur in developing countries, which have only 5% of the world’s cancer resources.^3^ Cervical cancer is the second most common cancer among women in the developing world and the most common cause of cancer deaths.[11] In the three countries in this study, cervical cancer rates are high. In Côte d’Ivoire the incidence of cervical cancer is 21.7/100,000 women and the mortality rate from cervical cancer is 14.6/100,000 women. Incidence and mortality rates are even higher in Guyana (46.9/100,000 and 21.9/100,000, respectively) and Tanzania (54.0/100,000 and 32.4/100,000, respectively). In contrast, in North America, where cervical cancer screening is routine, the cervical cancer incidence and mortality rates are only 6.6/100,000 and 2.7/100,000, respectively.[12]

Case-control and cross-sectional studies in various African countries, including Côte d’Ivoire and Tanzania, have found an association between HIV infection and invasive cervical cancer.[13,14] The strength of the association has varied in these studies, possibly reflecting the competing risk of dying from other HIV-related conditions or other illnesses.[12,15] HIV prevalence in Côte d’Ivoire (3%), Guyana (1.1%), and Tanzania (5.8%) is significantly higher than the overall HIV prevalence in North America (0.5%).[16] Unlike other typical opportunistic infections, the burden of HPV or HPV-related complications has not been shown to decline with effective ART;[17] this could mean that increased numbers of women will be at risk for cervical cancer as HIV treatment programs become more accessible and successful and HIV-positive women live longer. A recent mathematical model projected that, compared with no ART and no screening, the lifetime cumulative risk of dying from cervical cancer approximately doubled with ART and no screening. However, even one screening had the potential to reduce cervical cancer mortality.[18] These findings have important implications for lower-resource countries, where an increasing number of HIV-infected women are currently accessing HIV care and treatment.

Prevention of cervical cancer by identification and treatment of cervical cancer precursors is central to reducing the disease burden, because treatment resources for invasive diseases are scarce. In 2002, the survival rate for invasive cervical cancer was 21% in sub-Saharan Africa, likely related to late presentation and lack of effective treatment resources, including surgical expertise and radiotherapy.[19,20] However, cervical cytology, which has revolutionized cervical cancer prevention in developed countries, is not feasible for most countries with limited resources. Furthermore, many women in these settings reside at some distance from health centers and have little access to transportation. This is coupled with a lack of effective recall mechanisms for women with abnormal results.[7] In sub-Saharan Africa, loss-to-follow-up rates of 60–80% have been reported among those with cytologic abnormalities.[21]

Recent studies have focused on service delivery models using alternatives to cytology for cervical cancer screening in order to improve access to safe and effective treatment, minimize loss to treatment follow-up, and prioritize use of specialized care. VIA has been shown to be effective, safe, feasible, and acceptable in multiple studies.[9,22,23] It is inexpensive, can be task-shifted to lower-level health workers,[24] and allows screening and treatment in a single visit for those women that are eligible.

In a cluster randomized trial in India in which more than 31,000 women were followed for seven years, VIA with same-visit cryotherapy for positive results was associated with a 24% reduction in the incidence of stage 2 or higher cervical cancer and a 35% reduction in cervical cancer mortality.[9] A recent review of published studies of VIA accuracy, with histology as the standard and cervical intraepithelial neoplasia 2 (CIN 2 or high-grade cervical dysplasia) or higher as the outcome measure, found sensitivity to be 79–82%, specificity to be 91–92%, and positive predictive value to be 9–10%.[25] The World Health Organization (WHO) recently reported results from a cervical cancer screening demonstration project with VIA and SVA with cryotherapy in six African countries from 2005−2009. More than 19,000 women were screened; 10.1% were VIA positive and of these 87.7% were eligible for cryotherapy. The SVA enabled 39.1% to be screened and treated on the same day.[26]

Data on the accuracy of VIA in the setting of HIV infection are more limited. Studies comparing VIA to cytology, with biopsy-documented CIN 2 or higher as the standard, found that VIA equaled or outperformed cytology. In a research study of 1,202 HIV-infected women in South Africa, sensitivity for detection of CIN 2 or higher was the highest for HPV testing (92%) with 76% for both cytology and for VIA (with physician interpretation); specificity was lowest for HPV testing (51%) and highest for cytology (83%), with VIA intermediate at 68%. When the results of two tests were combined as either test positive, the HPV/VIA combination achieved the highest sensitivity at 95.6%, while the cytology/VIA combination had the highest specificity at 60.4%.[27] Among 1,128 HIV-infected women screened in India, sensitivity and specificity for VIA was 83.6% and 88.8%, respectively, as compared to 63.3% and 94.5% for cytology, respectively.[28]

Reports from Kenya, Botswana, Zambia, and Côte d’Ivoire demonstrate that integration of VIA into routine HIV care and in settings with high HIV prevalence is feasible and acceptable [29,30,31,32]. The program in Zambia had the largest number of patients and longest experience, screening 56,247 women over five years, approximately 15,000 of whom were HIV-infected, and demonstrating the successful incorporation of VIA screening and SVA through an HIV-care platform. The study showed that with increasing experience, providers performed more procedures accurately; rates of VIA positivity fell over time regardless of HIV status. [29] In a screen and treat program in Western Kenya, of 1,331 HIV-infected and HIV-uninfected women who were VIA positive, the overall loss to treatment follow up was 31.5%; rates increased as invasiveness of treatment increased.[33] In one study in North American, the loss to treatment follow-up rate from abnormal Pap smear results was 26%,[34] demonstrating the value of SVA, which is not available with cytology.

A randomized clinical trial of VIA and HPV testing, with cryotherapy treatment for positive results, was performed among more than 6,500 women in South Africa, of whom 956 were HIV-infected. Thirty-six months after screening, women were followed up with colposcopy and biopsy to determine the presence of CIN 2. VIA reduced the likelihood of CIN 2 at follow-up, but to a lesser degree than HPV testing, and statistical significance was attained only in HIV-infected women (RR 0.51 [95% CI; 0.29–0.89]).[35]

The evaluation reported in this study has several limitations. HIV testing was only performed in the context of the cervical cancer screening program in Tanzania for those women who reported HIV status unknown or had a negative result older than six months and at times test kits were not available; we were therefore largely dependent on self-report of HIV status, especially in Guyana, where the majority of screening was performed in reproductive and child health clinics. However, false-positive reports of HIV infection are likely uncommon, given stigma and discrimination associated with HIV status. It is likely that some of the women in the HIV-uninfected/unknown comparison group were in fact HIV-infected. If this is true, it is probable that misclassification resulted in an underestimation of the differences between the HIV-infected and the HIV-uninfected/unknown groups. Sensitivity analysis performed on Tanzanian data treating those with unknown HIV status as either HIV-positive or HIV-negative did not change findings (data not shown). Because this analysis was conducted within existing private and public sector clinics and in a “real world setting” and not in the context of a research study, there may be possible sources of bias. Providers were not blinded to the HIV status of the women they screened, possibly leading to over-call of VIA positivity in HIV-infected women. The finding that women in Guyana had higher odds of being VIA-positive may reflect the inherent subjectivity of VIA or other individual or country specific factors not captured in this evaluation. Additional potentially confounding variables such as age, CD4 count, and ART status were not available for this analysis. Although VIA-positive findings were not confirmed by biopsy in this program evaluation, studies noted above have validated the performance characteristics of VIA.

The findings of this analysis from a real-world setting are consistent with results of research studies in well-resourced settings using cytology as a screening technique and studies from low resource setting using VIA as the primary screen, which have found that HIV-infected women have higher rates of cervical precancerous abnormalities compared to HIV-uninfected women. The strengths of this evaluation include the confirmation that HIV-infected women are more likely to have lesions involving a larger area of the cervix, requiring more extensive treatment, and establishment of SVA as a strategy to reduce loss to treatment follow-up for cryotherapy-eligible women. The large number of women screened and the consistency of primary findings across countries in both South America and West and East Africa, despite limitations in available data, as well as the inclusion of varied sites and different clinical settings, enhance the validity of our findings.

## Conclusions

An overarching goal of the new WHO global health sector strategy on HIV/AIDS is to achieve universal access to comprehensive HIV prevention, treatment, and care, including strengthening linkages between HIV and other related health programs, such as cervical cancer screening and care.[36] The findings of this study have significant implications for the integration of cervical cancer screening into HIV care. They suggest an increased need for referral and/or incorporation of excisional treatment, rather than cryotherapy alone, requiring more training and greater resources. Further analyses that include ART status and CD4 counts are important to further inform screening and treatment protocols and guidelines on appropriate screening strategies and intervals. Further study is needed to assess whether screening earlier in the course of HIV, when there is less immunosuppression, might also be associated with smaller and more treatable lesions, or whether ART and associated immune reconstitution would make a difference in rates of VIA positivity and lesion size. It is also critical to consider models of care to integrate cervical cancer screening into HIV care settings to enhance test performance, feasibility, and scale-up.
